# Increased Toll-Like Receptor Signaling Pathways Characterize CD8+ Cells in Rapidly Progressive SIV Infection

**DOI:** 10.1155/2013/796014

**Published:** 2012-12-27

**Authors:** Maria Cecilia Garibaldi Marcondes, Celsa Spina, Eduardo Bustamante, Howard Fox

**Affiliations:** ^1^Molecular and Integrative Neurosciences Department, The Scripps Research Institute, La Jolla, CA 92037, USA; ^2^Department of Pathology, UCSD School of Medicine, AIDS Research Center, La Jolla, CA 92037, USA; ^3^Department of Pharmacology and Experimental Neuroscience, University of Nebraska Medical Center, Omaha, NE 68198, USA

## Abstract

Similar to HIV infection in humans, SIV infection in macaques induces progressive loss of immune cell components and function, resulting in immune deficiency in nearly all untreated infected subjects. In SIV-infected macaques, 25% of animals develop terminal AIDS within 6 months of infection. The factors responsible for the development of such rapid progression are unknown. We have previously found that defects in CD8+ T cells detectable from early infection correlate to rapid progression to simian AIDS. The transcriptional screening of molecular fingerprints on different steps along the activation/effector process of splenic CD8+ cells at termination revealed a distinction in rapid compared to regular progressors, which was characterized by a decrease in classic T cell receptor (TCR) components, and an increase in Toll-like receptor (TLR) and apoptotic pathways. A TLR pathway screening in lymphoid and myeloid cells from both the spleen and from the central nervous system of infected macaques revealed that the upregulation of TLR is not in the innate immune compartment, but rather in lymphoid cells that contain adaptive immune cells. Our findings suggest that opposing effects of TCR specific signaling and TLR engagement may drive the CD8 phenotypic failure that determines a rapid disease course in HIV infection.

## 1. Introduction

SIV infection in macaques and HIV infection in humans follow a similar pattern. The SIV-infected rhesus macaque model has been useful for studying many aspects of HIV pathogenesis. One such finding was a crucial role for CD8+ cells, where their acute depletion in the early infection period leads to high viremia and rapid progression [1–3]. Even in the absence of this manipulation, rapid progression (≤200 days) occurs in 25% of SIV infected animals [4–6]. Hallmarks of rapid progressing (RAP) animals include a low antibody response to the virus [7], fatal immunodeficiency linked to deficits in tissue-homing memory CD4 cells [8, 9], and a severe central nervous system (CNS) disease characterized by encephalitis [4, 10]. 

Little is known on how the CD8 performance influences the CD4 decline in spontaneous rapid progression. Poor antivirus CD8+ cytotoxic T lymphocyte (CTL) response was shown in RAPs [11–14], both in SIV and HIV. CTLs become defective regardless of epitope escape [10, 15–17]. In RAPs, known peptide-specific CTL populations collapse along with a decrease of activation/memory markers in all CD8+ cells, and a loss of memory CD4 cells [10]. Likewise, experimental induction of CD8 collapse using depleting antibodies (as above) leads to loss of memory/activated CD4 cells [10]. These observations suggest that dysfunctional CD8+ cells could contribute to disease progression in HIV-infected individuals. 

Deficient CD8+ cell responsiveness is a potential cause of uncontrolled virus replication, driving disease progression, and failure to maintain the CD4 memory pool. To test the basis of CD8+ cell collapse, we compared molecular changes triggered by the virus in splenic CD8+ cells isolated from RAPs and those that did not (regular progressors, REGs), with CD8+ cells from uninfected controls. Along key steps of the CD8 stimulation process (activation, regulation, and effector function), we have identified transcriptional alterations that may help understand CD8 functional deficits observed in correlation to rapid progression and AIDS. These alterations were mainly related to initial steps of the CD8 stimulation, which in REGs followed a typical T-cell receptor (TCR) specific engagement pattern, but in RAPs, a TLR-triggered pathway was rather activated. 

Given that Toll-like receptors (TLRs) have been extensively proposed as adjuvants for HIV vaccination approaches, we determined that it was important to perform an in-depth investigation of TLR expression pattern and associated coadaptors in correlation to rapid progression. We also examined resulting activation pathways at the transcriptional level both in the innate and in the adaptive immune compartments of SIV-infected macaques exhibiting the accelerated development of AIDS, in comparison to animals that follow a more chronic course. We further confirmed the TLR activation to occur predominantly in the adaptive T-cells compartment, by comparing the expression of TLR pathway components in lymphoid cells in comparison to the myeloid from the spleen. Our results suggest that TLR engagement and inefficient virus-specific TCR signaling are linked to CD8 phenotypic characteristics of rapid progression in HIV infection. The overexpression of TLRs may be predictive of disease outcome as a marker of hyperactivation in the absence of effective specific T-cell response, and potentially constitute a marker of immune senescence.

## 2. Material and Methods

### 2.1. Animals

 Rhesus macaques were infected with a stock derived from SIVmac251, containing 1.25 ng of p27 (gag) Ag. Animals 298, 332, and 357 were uninfected and sacrificed as controls. Animals 350, 353, and 354 were sacrificed at 73, 77, and 80 days after infection (p.i.), respectively, and comprised the 11 wk p.i. group (REG 11 weeks pi). Animals 417, 418, 523, and 529 developed signs of simian AIDS and were sacrificed at days 56, 82, 140, and 34 p.i., respectively (RAP). Some of these animals have been characterized in previous studies [10, 17, 18]. 

### 2.2. Cells

 At necropsy, splenic and brain cells were obtained and either were used for flow cytometry or culture, or cryopreserved as previously described [19]. Cells were thawed and washed in fetal bovine serum and were counted and incubated with anti-CD8 or anti-CD11b magnetic beads for magnetic separation and enrichment of CD8+ T cells using Miltenyi Biotech, Inc., separation system (Miltenyi Biotec, Auburn, CA, USA) following the manufacturer's protocol. The purity of cells was confirmed by flow cytometry as described [19], using non-overlapping anti-human CD8-PE (clone DK25; DAKO, Carpinteria, CA, USA) and anti-Mac-1-PE (clone M1/70, Roche, Indianapolis, IN, USA). Biotinylated anti-rhesus macaque CD3 (Biosource) and anti-human CD4 (L200 clone, BD Bioscience) were also used. Cells were acquired in a FACSCalibur using CellQuest software (BD Immunocytometry Systems, San Jose, CA, USA) and analyzed using FlowJo 6.2.1 software (Tree Star, Ashland, OR, USA). 

### 2.3. RNA and qRT-PCR

 RNA from CD8+ and CD11b+ cell pellets were extracted using Ambion Totally RNA kit (Life Technologies, Carlsbad, CA, USA). First strand kit (SABiosciences, Qiagen, Valencia, CA, USA) for cDNA synthesis was performed. Isolated CD8+-enriched cells were screened using a custom made PCR array (SABiosciences). CD11b-enriched and CD11b-depleted cells were analyzed using a pathway array analysis of rhesus macaque TLR pathways (RT2 Profiler PCR array, PAQQ-018C; SABiosciences). SyBr Green real-time PCRs were performed in ABI Prism 7900 HT instrument (Applied Biosystems) using ABI Prism 7900 SDS 2.1. For gene expression, raw data threshold was normalized using GAPDH or the average of 5 housekeeping genes, as indicated at figures legends, yielding dCt values.

### 2.4. Statistical Analysis

The screening of CD8 functional performance was made on 48 genes and analyzed using one-way ANOVA and post hoc Tukey's tests, using Prism (GraphPad Software). The TLR pathway analysis on sorted cells was performed on 86 genes and comparison between groups was performed using Student's *t*-test, using the web-based analysis tool PCR Array Data Analysis Web Portal (http://www.sabioscience.com/). 

## 3. Results

### 3.1. RAPs Have TCR-Mediated Signaling and Several Functional Molecules at Low Levels but Highly Upregulated TLR Coadaptor MyD88 in CD8+ Cells

**Figure 1:**
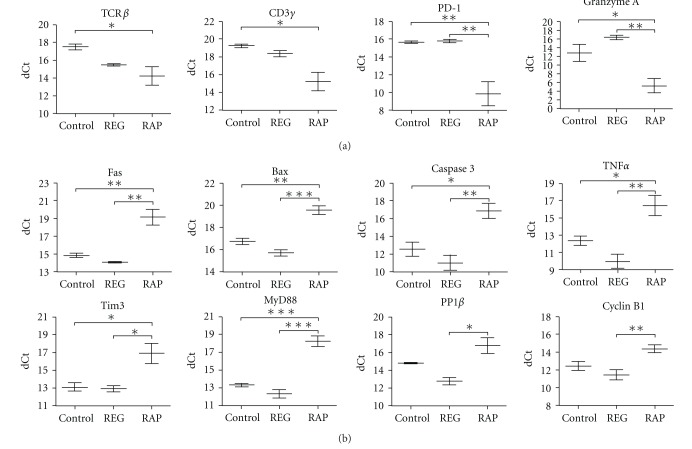
Gene expression in CD8+ cells in uninfected and SIV infected REG and RAP monkeys. The expression of activation molecules was normalized against GAPDH. (a) Genes decreased in RAPs. Multiple group significances demonstrated by one-way ANOVA (TCR*β* = 0.0483, CD3*γ* = 0.0149, PD-1 = 0.0044, granzyme A = 0.0033). (b) Genes increased in RAPS. Multiple group significances demonstrated by one-way ANOVA (Fas = 0.0015, Bax = 0.0002, Caspase 3 = 0.0034, TNF*α* = 0.0056, Tim3 = 0.0180, MyD88 = 0.0001, PP1*β* = 0.0114, Cyclin B1 = 0.0099). For both (a) and (b) significance between the groups was determined by post hoc Tukey testing, and indicated by  *P < 0.05,  **P < 0.01, ^  ∗∗∗^ *P* < 0.001.

SIV-infected RAP macaques have poor antiviral CD8+ response and fail to maintain activation [10]. To understand the nature of the CD8 collapse, we have analyzed changes in splenic CD8+ cells from rhesus macaques infected with a SIVmac251-derived stock. Three groups were examined: (1) REGs at 11 weeks p.i., (2) RAPs at an average of 11 weeks p.i., and (3) uninfected controls. We first performed a screening for expression of key activation and functional molecules (Figure 1). For that, pure CD8+ cells were isolated from the spleen, using magnetic bead-labeled antibodies [19]. The purity of the CD8+ cell isolates by FACS was ≥95%; mRNA was extracted and reverse transcribed. Using quantitative PCR we compared the control and infected animals, grouping the infected monkeys by different progression patterns. 

Interestingly, of the 48 gene transcripts examined, 12 showed significant (*P* < 0.05) differences between the groups by ANOVA. These fell in two main categories. Post hoc Tukey testing revealed that four transcripts were downregulated in the RAP animals when compared to REG animals and/or the uninfected controls (Figure 1(a)). These comprised key components of CD8+ T cells and were strongly downregulated relative to the uninfected controls. TCR*β* and CD3*γ* are critical components of the antigen recognition and signaling process in CD8+ T cells, whereas granzyme A is part of the cytotoxic arsenal. While their downregulation can certainly compromise function, the downregulation of PD-1 (programmed cell death-1) may result in a reduction of CD8+ inhibitory functions. On the other hand, PD-1 downmodulation could also be a compensatory mechanism in an attempt to prevent CD8 exhaustion. 

Eight of the transcripts were significantly upregulated in RAP animals (Figure 1(b)). Six of these were significantly and strongly increased relative to both the uninfected controls and the REG animals, and three can be directly linked to apoptosis: Fas, Bax, and Caspase 3. Another Tim3 represents an inhibitory molecule, although in contrast to the PD-1 inhibitory molecule found downregulated above it is highly upregulated. TNF-*α* expression is also markedly increased, but neither other cytokines were examined (IFN-*γ*, IL2, IL17, IL21) nor cytokine receptors (IL2R*α*, IL7R, IL15R*α*, IL21R) were altered. The sixth molecule upregulated relative to both REGs and uninfected controls was MyD88. This increase was striking and intriguing given the key role of MyD88 in linking TLR recognition to NF-*κ*B activation. In fact, the induction of TNF-*α* is one of the major effects of such activation. Two additional transcripts were found increased in the RAPs relative to the REGs, the serine/threonine phosphatase PP1*β* and Cyclin B1, both of which are involved in cellular proliferation.

### 3.2. TLR Activation Is Concentrated in the Adaptive Immune Compartment

The decrease in TCR*β*, CD3*γ*, and granzyme A and increase in Tim3 are consistent with the lack of adaptive immune function in RAP CD8+ cells that we found in a previous study [10]. Tim3, in particular, has been shown to reduce cytotoxicity in exhausted CD8 cells during HIV infection [20], suggesting that the elevation of Tim3 in rapid progressors is consistent with a phenotype of exhaustion within the CD8+ compartment. 

**Figure 2:**
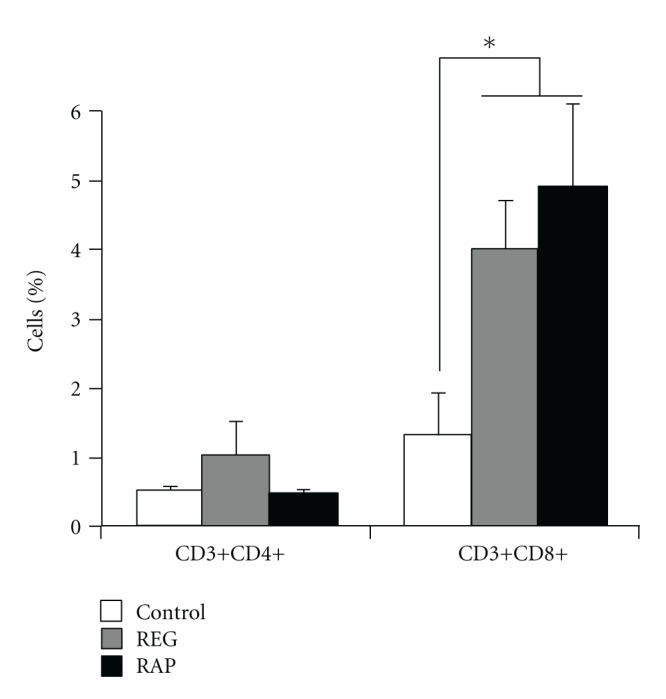
Percentage of T cells in the brain of controls and SIV-infected rhesus macaques exhibiting REG or RAP disease course. Brain cell suspensions were stained with fluorescent-labelled antibodies against CD3, CD4, and CD8 surface markers to identify CD3+CD4+ T helper cells and CD3+CD8+ cytotoxic T cells, using flow cytometry.  *P < 0.05 in comparison to uninfected control animals.

The strong increase in MyD88 along with the increased TNF*α* was consistent with potential activation of the TLR pathway. Since the differences between RAP and REG CD8+ cells were identified among initial activation molecules, we hypothesized that antigen presenting cells could be involved in the TLR activation. We thus examined the innate myeloid mononuclear cells, which include APC, from the splenic cells by selecting for CD11b+, as well as the CD11b− cells mainly consisting of T and B adaptive immune cells, for the expression of 84 genes related to the TLR signaling pathway and innate immunity. Strikingly the differences between RAP and REG were almost exclusive in the CD11b− lymphoid adaptive immune compartment (Figure 2), with significant increases in RAP animals in three of the TLRs (TLR3, 6, and 9) and multiple TLR adapter and interacting proteins, as well as the downstream NF*κ*B and JNK/p38 signaling pathways (Table 1). These include, as identified above, increases in MyD88 and TNF*α*. In contrast, in the CD11b+ myeloid cells, only one gene was significantly different between the RAPs and REGs, TLR10 (2.61-fold, *P* = 0.0343).

### 3.3. Isolated Brain Immune Cells from RAP Animals Also Show a Predominance on TLR-Mediated Response

RAP animals have a high incidence of SIV encephalitis (SIVE). While SIV-specific CD8+ T cells are present in the brains of these animals, they are ineffective in controlling the high brain viral load characterizing SIVE [10]. Similar proportions of CD8+ T cells are indeed present in brains of both REGs and RAPs (Figure 2). In order to ascertain whether the TLR pathways are also elevated in the CNS, we isolated CD8+ CNS infiltrating cells and examined them for expression of the TLR components using quantitative real-time PCR. Indeed five TLRs (TLR 1, 2, 3, 8, and 9) as well as, similar to the spleen lymphocytes, multiple TLR adapter and interacting proteins were upregulated in RAP animals. The downstream NF*κ*B and JNK/p38 signaling pathways are increased; however, we note that some were decreased, including TLR4 (Table 2). Overall, most examined molecules were increased, and, as found in the spleen, significant increases in MyD88 and TNF*α* were observed in the CD8+ cells in the brains of RAPs. 

Overall, we observed that rapid progression is characterized by a strong MyD88-dependent TLR activation in the absence of an effective anti-SIV specific response, causing an unbalance that can lead to exacerbated inflammatory response and lack of control over viral load. This was observed both in the spleen and in the CNS. 

## 4. Discussion

Rapid progressors have low or absent virus-specific CD8+ pool in the spleen and brain, as accessed by the number of Gag and Tat-specific cells, confirming previous findings [10]. This reduction may be on the base of a low TCR signaling and activation through nonspecific pathways such as TLR, generating rather bystander CD8+ cells, activated to become proinflammatory but not efficient against the infection. Here we show that the expression of TLRs is increased on splenic CD8+ cells from RAP animals in comparison to controls. Downstream adaptor molecule MyD88 was increased in RAPs, suggesting that in rapid progression upregulation of TLRs is followed by its engagement. The increase of TLR9 in splenic CD8+ cells from RAPs compared to REGs was validated by detection of intracellular levels by FACS (not shown).

These results put forward a potential mechanism for previous results from rapid progressors expressing MamuA*01 class I haplotype. When compared to regular MamuA*01+ progressors, not only they showed a reduced Tat and Gag-specific pool [10], but their CD8+ T cells failed to proliferate specifically in response to Gag CM9 and Tat SL8 peptides in vitro.

The initial failure of the TCR signaling originated from the absence of a specific pool and predominance of TLR pathways results in differences on performance and regulation of survival and cell cycle molecules, favoring the upregulation of molecules related to apoptosis. Indeed, direct TLR stimulation on T cells may result in increased apoptosis [21]. TLR2, in particular, which was upregulated both in the spleen and in the brain of RAP animals in comparison to REGs, can trigger MyD88-mediated apoptosis, involving Fas and Caspase 8 [22], depending on levels of Bcl-2 family molecules of pro- and antiapoptotic proteins [23]. We found upregulation of Fas and Caspases as well as decrease in Bcl-2 in splenic CD8+ cells from RAP animals. 

Several studies suggest that the action of TLR in CD8 activation is indirect and may require the participation of innate immune cells [24–26]. In the context of infection, CD8+ cells express TLR, but HIV does not infect these cells. Nevertheless, TLR agonists can directly stimulate CD8+ T cells [21, 27, 28]. We analyzed the performance of isolated CD11b cells from REGs and RAPs regarding the expression of TLRs and its adaptors. However, only TLR10 showed a significant upregulation in correlation with poor disease outcome in the innate compartment. This is interesting since the role of TLR10, which is mostly expressed by B cells and DCs [29], is not clear. 

Even though TLRs have been proposed as adjuvants in HIV vaccination approaches. For example, stimulation of TLR4, TLR2, TLR6, TLR7, and TLR8 has been used to enhance HIV-1-specific cellular, humoral, and mucosal immunity [30]. CpG ODNs, TLR9 ligands, are also effective adjuvants for peptide-based HIV-1 vaccines [31]. The magnitude and quality of the CD8+ response to HIV is improved when antigens are conjugated with TLR7/8 agonists [32, 33]. However, even in the absence of high affinity TCR engagement, TLR7 agonists can induce activation of CD8+ T cells [21], suggesting that virus- specific CTLs in the presence of synthetic agonists may be improved if the development of an antigen-specific pool precedes the TLR stimulation. 

Importantly, the response triggered by TLR engagement is not necessarily protective. Other studies have revealed a controversial role for TLRs in vaccination approaches. For instance, in a mouse model of HIV-1 infection, sustained TLR7 stimulation causes activation and disruption of the lymphoid system, leading to pathology and poor outcome [34]. Interestingly, data from SIV/macaque model provide additional support for a role of TLR pathways in HIV-1 pathogenesis. SIV infection in rhesus macaques induces strong immune activation and pathogenesis while in sooty mangabeys, the natural host of SIV, immune activation is significantly lower while disease is not observed. Conversely, stimulation with SIV, TLR7, or TLR9 ligands induced much stronger IFN production by pDCs derived from rhesus macaques compared with pDCs from sooty mangabeys. This happens in close correlation with genetic differences in IRF-7 between these 2 species, which could offer a potential explanation for differences in pDC responses to ligands [35]. TLR polymorphisms have been also found in humans in association with both HIV progression and protection [36]. 

In another study, monkeys intravaginally treated with TLR7 and TLR9 agonists prior to SIV challenge had significantly higher set point viremia than control animals that were PBS-treated [37], suggesting that the activation induced by direct TLR engagement on CD8+ cells is not enough to produce efficient antiviral response. Furthermore, on CD4 cells, it may enhance vaginal SIV transmission or posttransmission-SIV replication. Thus, TLR engagement ahead of the development of a virus-specific pool can generate poor CD8 performance characteristic of rapid progression [10]. 

Our results show that a failure in controlling viral load, both peripherally and in the brain, is strongly associated to a poor TCR-mediated response, lack of development of antiviral response, and characteristics of exhaustion in the CD8 compartment. In addition, immune cells from rapid progressors show instead a robust activation of MyD88-mediated TLR pathways, including downstream NF*κ*b and JNK/p38 pathways, and lead to upregulation of cell death markers. Our results suggest that there is a high risk in stimulating TLR response ahead of the development of a strong antiviral response. The use of TLR as vaccine adjuvants can be positive but it should be used with caution, in individuals where signs of specific response are detectable. In addition, hyperactivation of TLR pathways can be a strong predictive of rapid progression.

## Figures and Tables

**Table 1:** Comparison of expression levels of TLR signaling pathway and innate immunity genes in SIV-infected REG and RAP CD11b-depleted splenic cells. Expression levels were normalized using the average of 5 housekeeping genes and the ratio between RAP and REG animals' dCT values was calculated issuing fold change. Shown are the genes whose expression is significantly changed between the groups, organized by functional categories of the TLRs and their adapter as well as interacting proteins, the downstream NF*κ*B and JNK/p38 signaling pathways, as well as other related molecules (MAPK8IP3 is present in both the adapter/interacting and the JNK/p38 categories).

TLRs and adapter/interacting	Fold RAP/REG	*P* value	NF*κ*B & JNK/p38	Fold RAP/REG	*P* value	Other	Fold RAP/REG	*P* value
GPC1	9.43	0.0168	ESCIT	10.05	0.0235	CCL2	13.42	0.0193
HRAS	5.72	0.0061	IL10	12.73	0.0497	CD86	8.47	0.0216
LY86	9.96	0.0455	IRAK2	8.07	0.0121	CSF3	12.03	0.0161
MAL	10.28	0.0028	IRAK4	11.15	0.0310	IL6	11.17	0.0239
MAPK8IP3	12.26	0.0031	IRF3	9.74	0.0077	IRF1	9.23	0.0006
MYD88	12.39	0.0450	JUN	11.35	0.0387	IRF7	14.37	0.0412
PGLYRP3	12.08	0.0388	MAP2K3	11.65	0.0229	LTA	10.12	0.0196
SARM1	7.38	0.0335	MAP2K4	8.80	0.0282	PRKRA	12.03	0.0258
TIRAP	7.53	0.0275	MAPK12	10.05	0.0077	TBK1	10.79	0.0476
TLR3	13.90	0.0142	MAPK8IP3	12.26	0.0031			
TLR6	4.10	0.0472	NFKB2	8.70	0.0317			
TLR9	5.60	0.0489	NFKBIB	12.67	0.0242			
TOLLIP	4.70	0.0082	REL	6.45	0.0270			
			RELB	9.45	0.0401			
			TNF	9.51	0.0034			
			TNFRSF1A	8.12	0.0050			

**Table 2:** Comparison of expression levels of TLR signaling pathway and innate immunity genes in SIV-infected REG and RAP CD8+ cells isolated from the brain. Expression levels were normalized using the average of 5 housekeeping genes and the ratio between RAP and REG animals' dCT values was calculated issuing fold change. Shown are the genes whose expression is significantly changed between the groups, organized by functional categories of the TLRs and their adapter as well as interacting proteins, the downstream NF*κ*B and JNK/p38 signaling pathways, as well as other related molecules.

TLRs and adapter/interacting	Fold RAP/REG	*P* value	NF*κ*B & JNK/p38	Fold RAP/REG	*P* value	Other	Fold RAP/REG	*P* value
HRAS	1.43	0.0436	IL10	2.56	0.0373	CCL2	27.49	0.0338
MYD88	2.55	0.0183	IRF3	0.26	0.0123	IL12B	0.17	0.0387
PELI2	0.41	0.0011	MAP2K3	1.62	0.0400	IL2	3.38	0.0075
TLR1	1.95	0.0437	MAP4K4	0.46	0.0040	IRF1	3.08	0.0016
TLR2	4.60	0.0429	MAPK10	1.38	0.0374	IRF7	4.91	0.0009
TLR3	1.11	0.0417	NFKB1	1.65	0.0151	LTA	1.27	0.0245
TLR4	0.51	0.0483	NFKBIB	2.03	0.0300	PTGS2	2.57	0.0371
TLR8	2.64	0.0238	NFKBIE	1.37	0.0496	TBK1	1.20	0.0276
TLR9	1.71	0.0242	PPARA	0.44	0.0489			
TOLLIP	1.78	0.0376	REL	1.88	0.0032			
			TNF	4.47	0.0482			
			TNFRSF1A	2.72	0.0090			
